# Mortality Risk and Burden From a Spectrum of Causes in Relation to Size-Fractionated Particulate Matters: Time Series Analysis

**DOI:** 10.2196/41862

**Published:** 2023-10-09

**Authors:** Jun Yang, Hang Dong, Chao Yu, Bixia Li, Guozhen Lin, Sujuan Chen, Dongjie Cai, Lin Huang, Boguang Wang, Mengmeng Li

**Affiliations:** 1 School of Public Health Guangzhou Medical University Guangzhou China; 2 Institute for Environmental and Climate Research Jinan University Guangzhou China; 3 Guangzhou Center for Disease Control and Prevention Guangzhou China; 4 Institute of Public Health Guangzhou Medical University and Guangzhou Center for Disease Control and Prevention Guangzhou China; 5 Guangdong University of Science and Technology Dongguan China; 6 State Key Laboratory of Oncology in South China Guangdong Provincial Clinical Research Center for Cancer Sun Yat-sen University Cancer Center Guangzhou China

**Keywords:** size-fractionated particulate matter, cause-specific mortality, cardiovascular disease, respiratory disease, neoplasm, attributable burden

## Abstract

**Background:**

There is limited evidence regarding the adverse impact of particulate matters (PMs) on multiple body systems from both epidemiological and mechanistic studies. The association between size-fractionated PMs and mortality risk, as well as the burden of a whole spectrum of causes of death, remains poorly characterized.

**Objective:**

We aimed to examine the wide range of susceptible diseases affected by different sizes of PMs. We also assessed the association between PMs with an aerodynamic diameter less than 1 µm (PM_1_), 2.5 µm (PM_2.5_), and 10 µm (PM_10_) and deaths from 36 causes in Guangzhou, China.

**Methods:**

Daily data were obtained on cause-specific mortality, PMs, and meteorology from 2014 to 2016. A time-stratified case-crossover approach was applied to estimate the risk and burden of cause-specific mortality attributable to PMs after adjusting for potential confounding variables, such as long-term trend and seasonality, relative humidity, temperature, air pressure, and public holidays. Stratification analyses were further conducted to explore the potential modification effects of season and demographic characteristics (eg, gender and age). We also assessed the reduction in mortality achieved by meeting the new air quality guidelines set by the World Health Organization (WHO).

**Results:**

Positive and monotonic associations were generally observed between PMs and mortality. For every 10 μg/m^3^ increase in 4-day moving average concentrations of PM_1_, PM_2.5_, and PM_10_, the risk of all-cause mortality increased by 2.00% (95% CI 1.08%-2.92%), 1.54% (95% CI 0.93%-2.16%), and 1.38% (95% CI 0.95%-1.82%), respectively. Significant effects of size-fractionated PMs were observed for deaths attributed to nonaccidental causes, cardiovascular disease, respiratory disease, neoplasms, chronic rheumatic heart diseases, hypertensive diseases, cerebrovascular diseases, stroke, influenza, and pneumonia. If daily concentrations of PM_1_, PM_2.5_, and PM_10_ reached the WHO target levels of 10, 15, and 45 μg/m^3^, 7921 (95% empirical CI [eCI] 4454-11,206), 8303 (95% eCI 5063-11,248), and 8326 (95% eCI 5980-10690) deaths could be prevented, respectively. The effect estimates of PMs were relatively higher during hot months, among female individuals, and among those aged 85 years and older, although the differences between subgroups were not statistically significant.

**Conclusions:**

We observed positive and monotonical exposure-response curves between PMs and deaths from several diseases. The effect of PM_1_ was stronger on mortality than that of PM_2.5_ and PM_10_. A substantial number of premature deaths could be preventable by adhering to the WHO’s new guidelines for PMs. Our findings highlight the importance of a size-based strategy in controlling PMs and managing their health impact.

## Introduction

With the rapid socioeconomic development and fast urbanization, air pollution—particularly the particulate matters (PMs)—has become the biggest environmental challenge to human health globally [1]. According to the recent assessment of the global burden of disease, PMs accounted for over 4.1 million deaths [2]. As the largest developing country, China faces a disproportionately high health burden due to PM pollution [3]. The improvement of air pollution control policies warrants an in-depth knowledge and quantification of the health impact of PMs.

PMs consist of discrete particles that vary in size, which is an important characteristic influencing their hazardous effects. PMs with aerodynamic diameters less than 1 µm (PM_1_), 2.5 µm (PM_2.5_), and 10 µm (PM_10_) have been studied extensively due to their ability to enter and deposit in the respiratory tract. Previous epidemiological investigations have identified the harmful impact of PM_10_ and PM_2.5_ on human health [4,5]. In recent years, PM_1_ has also raised increasing concerns due to emerging evidence indicating higher health risks associated with PMs of smaller sizes [6-9].

Prior studies mainly focused on the impact of size-fractionated PMs on mortality from common chronic diseases (ie, circulatory and respiratory diseases) [1,3,4,6]. Although less explored, PMs have been linked to an increased risk of developing and dying from diabetes and pancreatic cancer [5,10]. The risk of dying from external causes, including intentional self-harm, was also associated with increased levels of PMs [11]. There is limited evidence to suggest that PMs might also affect other systems, such as digestive, nervous, and genitourinary systems [12,13]. Mechanistic studies have revealed that environmental exposures can cause oxidative stress and inflammation, genomic and epigenetic alterations, mitochondrial dysfunction, endocrine disruption, altered intercellular communication, altered microbiome communities, and impaired nervous system function [14]. We, therefore, hypothesize that size-fractionated PMs can increase the risk of death from diseases involving multiple human body systems, and this excess risk might vary depending on the size of PMs.

We performed a comprehensive evaluation of the relationship between size-fractionated PMs and cause-specific mortality using granular data collected from Guangzhou, a city in China highly polluted by PMs, with PM_1_ and PM_2.5_ concentrations reaching as high as 122 μg/m^3^ and 150 μg/m^3^ during our study period. Our aim was to examine the wide range of susceptible diseases affected by different sizes of PMs. Specifically, we examined deaths from 10 broad categories of causes (ie, all cause, nonaccidental, cardiovascular, respiratory, digestive, genitourinary, nervous system, endocrine system, external causes, and neoplasms), along with their 26 subcategories. These categories were assumed to be affected by PMs based on existing evidence, as mentioned earlier.

## Methods

### Health Data

Daily mortality data and weekly counts of influenza-like illness (ILI) for the years 2014-2016 were collected from the Guangzhou Center for Disease Control and Prevention. The causes of death were coded according to the International Classification of Diseases, Tenth Revision. All-cause and nonaccidental mortality were defined using the codes A00-Z99 and A00-R99 (from the International Classification of Diseases, Tenth Revision), respectively. We also extracted data regarding deaths due to cardiovascular (I00-I99), respiratory (J00-J99), digestive (K00-K93), and genitourinary diseases (N00-N99), together with diseases of the nervous system (G00-G99) and endocrine system (D50-D89 and E00-E90). Data on deaths from external causes (V01-Y89) and neoplasms (C00-D48) were also collected. In addition, 26 subcategories within the previously mentioned 10 broad disease categories were also considered (Table S1 in Multimedia Appendix 1). In addition, daily counts of deaths were further categorized by age group (≤64, 65-74, 75-84, and ≥85 years) and gender.

### Environmental Data

Daily concentrations of PM_1_ during the 2014-2016 period were collected from the monitoring stations of the Chinese Atmosphere Watch Network in Guangzhou [15,16]. Data for PM_2.5_ and PM_10_ were obtained from Guangzhou Bureau of Environmental Protection, together with data for other air pollutants, including ozone, sulfur dioxide, nitrogen dioxide, and carbon monoxide. We averaged the daily concentrations for each pollutant from 11 fixed-site monitoring stations in Guangzhou (Figure S1 in Multimedia Appendix 1). China Meteorological Data Service Center provided the daily meteorological data in Guangzhou, containing daily relative humidity (%); minimum, mean, and maximum temperatures (^o^C); and air pressure (hPa).

### Ethical Considerations

This study involved only a secondary analysis of daily aggregated and deidentified data, and it is classified as exempt from institutional review board approval according to the Chinese legal documents on ethics review issued by the National Health Commission of the People’s Republic of China (document number: 4; 2023) [17].

### Statistical Analyses

We conducted a time-stratified case-crossover method to evaluate the impact of PMs (PM_1_, PM_2.5_, and PM_10_) on mortality [11,18]. The following quasi-Poisson function accounting for overdispersion was used to assess the relationship between size-fractionated PMs and cause-specific mortality:



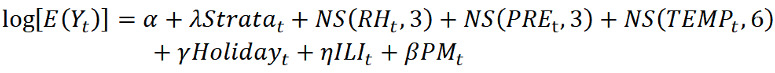



where *Y_t_* is the observed number of daily deaths on day *t*; α is the model intercept; *Strata_t_* denotes the time stratum variable used to control for seasonality and long-term trend; NS represents the natural cubic spline function, with 3 dfs for relative humidity (*RH_t_*) and air pressure (*PRE_t_*) and 6 dfs for mean temperature (*TEMP_t_*); *λ* , *γ*, and *η* are the vectors of coefficients. *β* denotes the changes in mortality risk per every 10 μg/m^3^ increase in the concentrations of PM pollutants [3,5,6]. Given that influenza could potentially confound the association between air pollution and health [19], the daily average of *ILI_t_* occurrence was also included in the model. Relative risk [*RR=exp(β)*] was estimated from the model and the impact of PMs was expressed as the percentage change [*(RR-1)* × *100%*] in the daily number of mortality associated with a 10 μg/m^3^ increment in PMs.

Furthermore, a smoothing spline function with 3 dfs was used to evaluate the exposure-response relationship between PMs (PM_1_, PM_2.5_, and PM_10_) and mortality risk. We also conducted a sensitivity analysis by changing the dfs (5-9) to test the robustness of the association. To investigate the lag patterns of PMs, we fitted the models using different single lags. Moving average approach was further applied to capture the cumulative lag effects of PMs.

To estimate the excess mortality burden caused by size-fractionated PMs with daily concentrations higher than the recommended target, we considered 5 target levels of PM_2.5_ and PM_10_ as per the World Health Organization’s (WHO) new air quality guidelines. The air quality guideline level and 4 interim targets for PM_2.5_ are 15, 25, 37.5, 50, and 75 μg/m^3^, respectively, while for PM_10,_ they are 45, 50, 75, 100, and 150 μg/m^3^, respectively [20]. There are no officially announced target levels for PM_1_ yet; however, given the high correlation between PM_1_ and PM_2.5_ and the fact that they are both secondary pollutants emitted from some common sources [21], we assumed the percentiles of the PM_1_ target levels to be the same as those of the PM_2.5_ target levels. We were able to identify the PM_1_ target levels (10, 20, 30, 40, and 55 μg/m^3^) by locating these percentiles within the PM_1_ distribution (Table S2 in Multimedia Appendix 1). The death burden due to PMs was calculated by combining the relative risk of PMs associated with their daily concentrations and the corresponding observed daily number of deaths. The total number of deaths attributable to each specific PM target was computed by summing the excess deaths when daily PM concentrations exceeded that target. The empirical CI (eCI) for the attributable deaths was estimated using Monte Carlo simulation with 1000 replications [5,22].

Stratification analyses were conducted to explore the potential modification effects of season (cold period: November-April; warm period: May-October) and demographical characteristics (gender and age) on the relationship between PMs and mortality. The *z* statistic was performed to test the statistical difference between the 2 relative risks obtained from the subgroup analyses [23,24].

### Sensitivity Analyses

The robustness of our main findings was examined by several analytical strategies. First, we changed the dfs for daily meteorological variables from 3 to 6. Second, we changed the dfs for the time variable from 3 to 9 per year. Finally, to test the confounding influence of other air pollutants, we performed the two-pollutant models by separately introducing a pair of air pollutants that are not highly correlated, thus avoiding collinearity (Spearman correlation coefficient <0.7) [3,25]. All the data analyses were conducted using the R software (version 4.0.1; R Core Team), and a 2-sided *P* value less than .05 was considered statistically significant.

## Results

### Summary of Descriptive Statistics

In total, there were 146,459 all-cause deaths from 2014 to 2016 in Guangzhou. The number of deaths was 138,396 for nonaccidental causes, including 56,587 circulatory deaths, 21,395 respiratory deaths, 4587 deaths from digestive diseases, 1303 from nervous system diseases, 1930 from genitourinary diseases, 8052 from external causes, 5469 from endocrine diseases, and 41,709 from neoplasms. The average concentrations of daily PM_1_, PM_2.5_, and PM_10_ were 28 (SD 13) μg/m^3^, 38 (SD 21) μg/m^3^, and 56 (SD 28) μg/m^3^, respectively (Table 1 and Table S1 In Multimedia Appendix 1). The correlation coefficients between PM_1_, PM_2.5_, PM_10_, and other air pollutants were less than 0.7, except for nitrogen dioxide. Temperature and relative humidity were negatively correlated with PMs, while air pressure was positively correlated with PMs (Table S3 in Multimedia Appendix 1).

**Table 1:** Summary statistics of environment and mortality data from 2014 to 2016 in Guangzhou, China.

Variables		Mean (SD)	Minimum, n	Percentile^a^					Maximum, n
				P5	P25	P50	P75	P95	
**Air pollutants**									
	PM_1_^b^ (μg/m^3^)	28 (16)	3	9	16	25	36	57	122
	PM_2.5_ (μg/m^3^)	38 (21)	6	15	22	33	48	77	150
	PM_10_ (μg/m^3^)	56 (28)	10	24	35	48	70	108	190
	Ozone (μg/m^3^)	78 (47)	4	16	42	72	107	163	254
	Sulfur dioxide (μg/m^3^)	13 (6)	3	5	9	12	16	22	38
	Nitrogen dioxide (μg/m^3^)	43 (17)	13	22	31	39	52	77	146
	Carbon monoxide (mg/m^3^)	0.9 (0.2)	0.4	0.6	0.7	0.8	1	1.3	2.5
**Meteorological variables**									
	Temperature (^o^C)	22 (6)	3	11	17	24	27	30	31
	Relative humidity (%)	79 (10)	31	62	74	80	87	93	98
	Air pressure (hPa)	1005 (7)	986	994	1000	1004	1010	1016	1028
**Daily deaths**									
	All cause	134 (23)	86	102	118	130	148	174	251
	Nonaccidental	126 (22)	80	96	110	124	140	166	238
	Circulatory disease	52 (13)	21	35	42	50	59	75	115
	Respiratory disease	20 (6)	6	11	15	19	24	32	47
	Digestive disease	4 (2)	0	1	3	4	5	8	12
	Nervous disease	1 (1)	0	0	0	1	2	3	7
	Genitourinary disease	2 (1)	0	0	1	2	3	4	8
	External causes	7 (3)	0	3	5	7	9	12	17
	Endocrine diseases	5 (2)	0	1	3	5	6	9	15
	Neoplasms	38 (6)	17	27	33	38	42	49	58

^a^P5, P25, P50, P75, and P95 denote the 5th, 25th, 50th, 75th, and 95th percentiles.

^b^PM_1_: particulate matters with an aerodynamic diameter less than 1 µm.

### Lag Pattern Effects of PMs on Mortality

**Figure 1:**
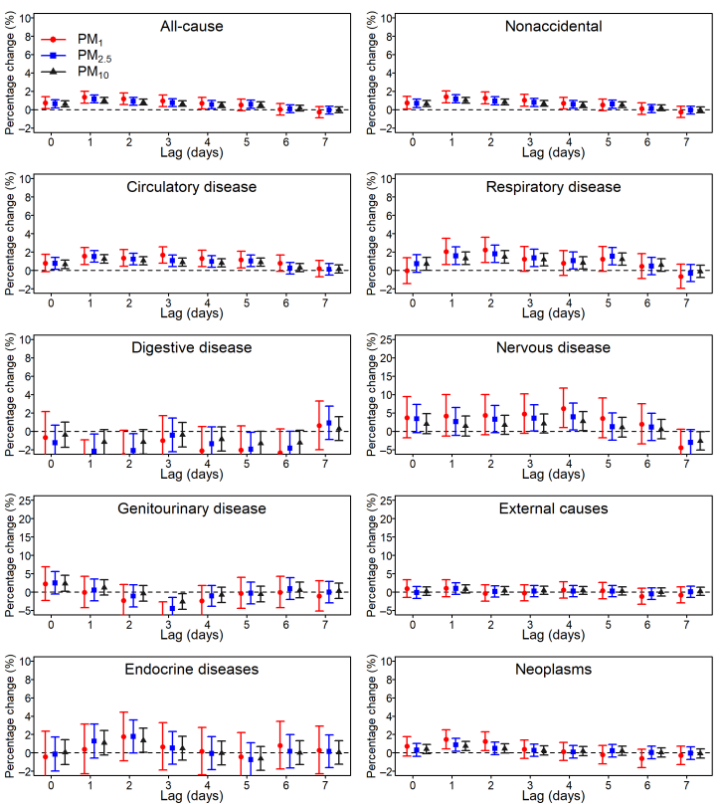
The percentage change (%) of cause-specific mortality associated with a 10 μg/m3 increase in size-fractionated particulate matters (PMs) on lag days 0-10. The vertical red, blue, and black lines denote the effect estimates of PM_1_, PM_2.5_, and PM_10_, respectively, on mortality across lag days.

The lag pattern effects of PM_1_, PM_2.5_, and PM_10_ on mortality risk manifested similar patterns, with estimates peaking at lag 1 or lag 2 for all-cause and nonaccidental mortality as well as for deaths from circulatory diseases, respiratory diseases, and neoplasms. The effects generally lasted for 4 days (Figure 1); however, the effects were statistically nonsignificant at different lag days for other diseases.

### Dose-Response Relationships Between Size-Fractionated PMs and Mortality

**Figure 2:**
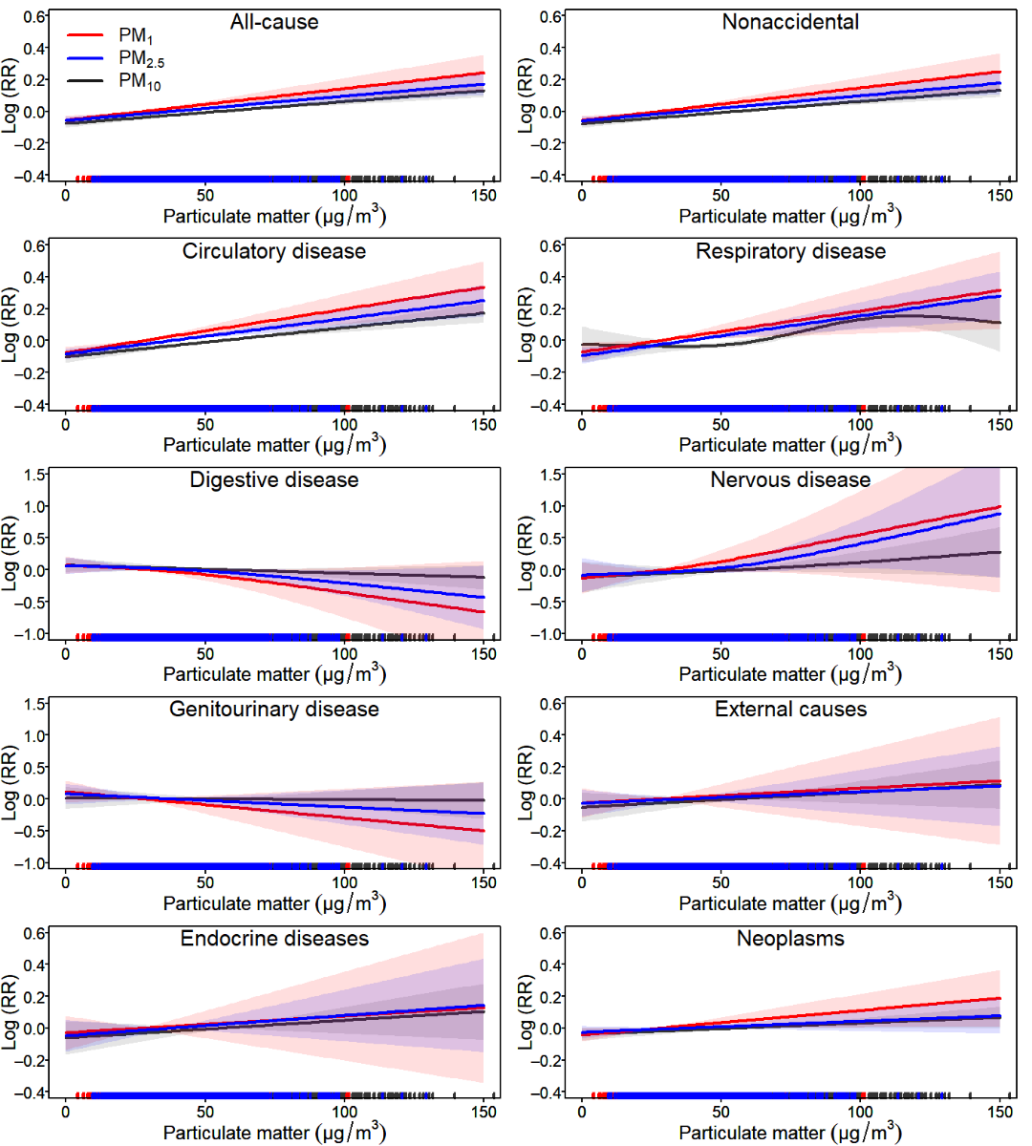
The concentration-response curves of size-fractionated particulate matters (PMs) and cause-specific mortality on lag days 0-3, using a 3-df smoothness for particulate matters. Red, blue, and black lines denote the PM_1_-mortality, PM_2.5_-mortality, and PM_10_-mortality associations, respectively; the shaded areas represent the 95% CIs. RR: relative risk.

The association between size-fractionated PMs and mortality at lag days 0-3 were generally positive and monotonically increasing (Figure 2). Similar associations were obtained when using 5-9 dfs for the spline function (Figures S2-S4 in Multimedia Appendix 1).

### Diseases Sensitive to Size-Fractionated PMs

**Figure 3:**
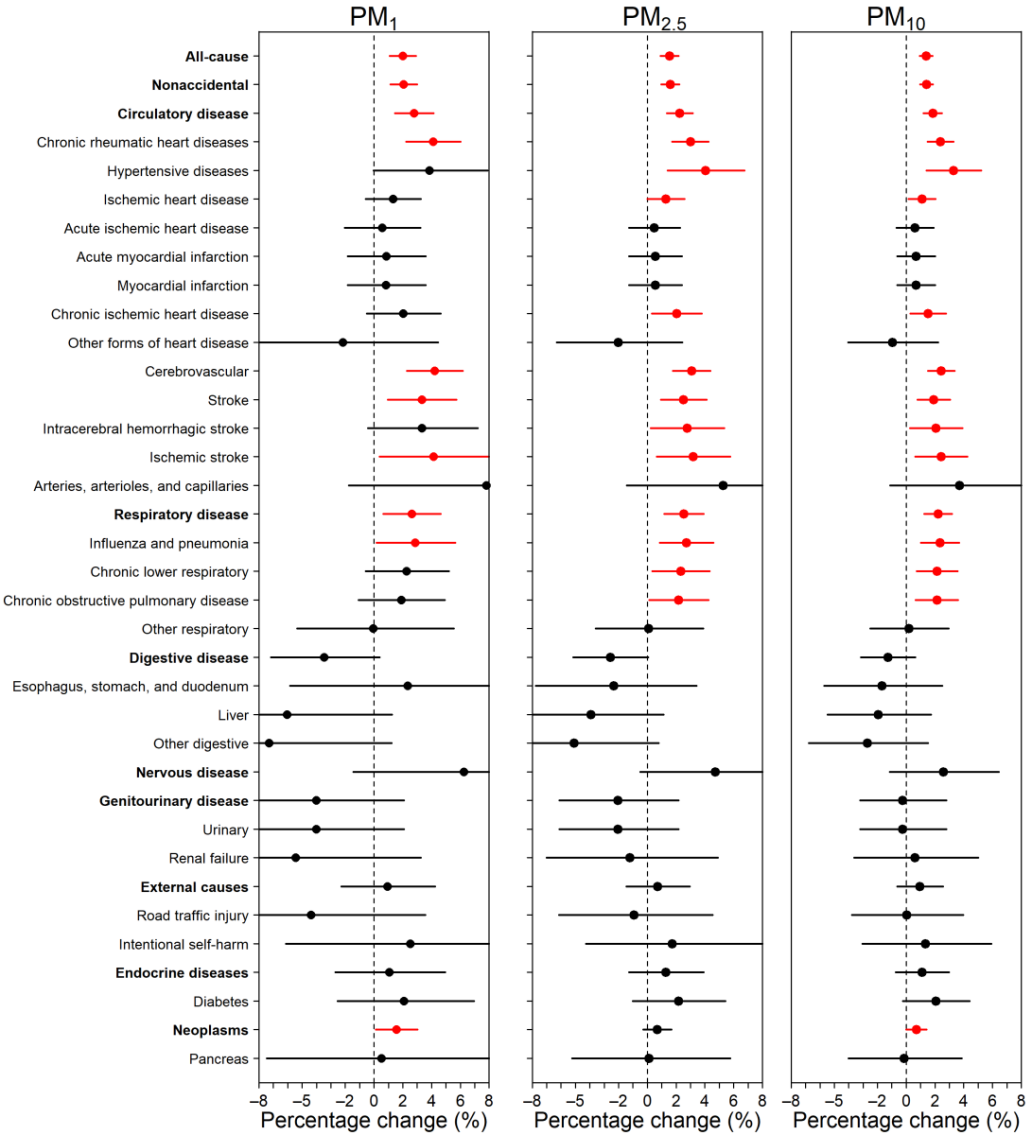
Percentage change (%) of cause-specific mortality per 10 μg/m3 increase in PM_1_, PM_2.5_, and PM_10_ on lag days 0-3. For each cause, point estimates (dots) and 95% CIs (horizontal lines) are shown. Red lines indicate statistically significant estimates. PM: particulate matter.

For every 10 μg/m^3^ increment in 4-day average concentrations of PM_1_, PM_2.5_, and PM_10_, the risk of all-cause mortality increased by 2.00% (95% CI 1.08%-2.92%), 1.54% (95% CI 0.93%-2.16%), and 1.38% (95% CI 0.95%-1.82%), respectively. The increments in nonaccidental mortality were similar across the 3 PMs (2.06%, 1.59%, and 1.41%). The largest effect estimates were observed for cardiovascular diseases (2.78%, 2.25%, and 1.85%) and respiratory diseases (2.61%, 2.53%, and 2.21%). The association between size-fractionated PMs and deaths from neoplasms were marginally significant, with effect estimates of 1.55% (95% CI 0.11%-3.01%), 0.69% (95% CI –0.28% to 1.67%), and 0.71% (95% CI 0.01%-1.40%; Figure 3 and Table S4 in Multimedia Appendix 1). The effect estimates of all size-fractionated PMs were statistically nonsignificant for deaths from nervous system diseases, digestive system diseases, genitourinary diseases, external causes, and endocrine diseases. For specific subcategories, significant effect estimates of PMs were detected among deaths due to chronic rheumatic heart diseases, hypertensive and cerebrovascular diseases, stroke (notably ischemic stroke), influenza, and pneumonia.

### PMs’ Effects on Mortality by Season and Individual Characteristics

In the subgroup analyses by season, age, and gender, we found a slightly greater impact of PM_1_ on mortality in hot months compared to cold ones. The mortality risk of PM_1_ was higher among older people, particularly for those aged ≥85 years. The effect estimates of PM_1_ were slightly stronger in female individuals compared to their male counterparts for overall and nonaccidental mortality and deaths from neoplasms. However, the opposite trend was observed for cardiovascular diseases and respiratory diseases (Table 2). A similar trend by seasons and individual characteristics was observed for PM_2.5_ and PM_10_ (Tables S5 and S6 in Multimedia Appendix 1). However, the differences in mortality risks of PM between subgroups (ie, season, age, and gender) were not statistically significant (*P*>.05).

**Table 2:** Percentage change in mortality per 10 μg/m3 increase in PM_1_ on lag days 0-3, stratified by season, age group, and gender.

Variables		Causes of death (%, 95% CI)				
		All cause	Nonaccidental	Cardiovascular	Respiratory	Neoplasms
Total		2.00 (1.08 to 2.92)	2.06 (1.13 to 3.00)	2.78 (1.46 to 4.13)	2.61 (0.63 to 4.63)	1.55 (0.11 to 3.01)
**Season**						
	Cold	1.66 (0.64 to 2.69)	1.74 (0.70 to 2.79)	2.14 (0.69 to 3.63)	2.56 (0.36 to 4.81)	1.29 (–0.35 to 2.96)
	Hot	3.11 (1.31 to 4.94)	3.13 (1.29 to 5.00)	5.18 (2.44 to 8.00)	2.79 (–1.20 to 6.95)	2.27 (–0.38 to 5.00)
**Age (years)**						
	0-64	1.22 (–0.24 to 2.70)	1.49 (–0.07 to 3.07)	2.21 (–0.83 to 5.34)	2.70 (–3.94 to 9.79)	0.61 (–1.46 to 2.72)
	65-74	1.50 (–0.32 to 3.35)	1.47 (–0.39 to 3.36)	1.74 (–1.21 to 4.78)	–0.87 (–6.20 to 4.75)	2.55 (–0.30 to 5.48)
	75-84	2.17 (0.73 to 3.63)	2.21 (0.75 to 3.69)	2.85 (0.84 to 4.90)	2.91 (–0.15 to 6.07)	1.04 (–1.58 to 3.74)
	≥85	2.83 (1.22 to 4.47)	2.77 (1.14 to 4.42)	3.45 (1.23 to 5.72)	3.28 (0.33 to 6.31)	4.87 (0.17 to 9.78)
**Gender**						
	Male	1.88 (0.81 to 2.97)	1.96 (0.86 to 3.08)	3.49 (1.81 to 5.20)	2.96 (0.41 to 5.57)	1.05 (–0.71 to 2.83)
	Female	2.16 (0.90 to 3.44)	2.20 (0.92 to 3.50)	2.07 (0.26 to 3.90)	2.15 (–0.73 to 5.11)	2.43 (0.04 to 4.89)

As presented in Table 3, the excess all-cause deaths advanced by PM_1_ were 7921 (95% eCI 4454-11,206), 6790 (95% eCI 3704-9611), 4899 (95% eCI 2696-6971), 3053 (95% eCI 1790-4393), and 1278 (95% eCI 711-1831), respectively, when the daily concentrations exceeded the target levels of 10, 20, 30, 40, and 55 μg/m^3^. For PM_2.5_, the excess death burden was 8303 (95% eCI 5063-11,248), 7194 (95% eCI 4183-9833), 5504 (95% eCI 3245-7620), 3717 (95% eCI 2242-5171), and 1352 (95% eCI 840-1861), with daily concentrations exceeding the WHO targets (15, 25, 37.5, 50, and 75 μg/m^3^), respectively (Table S7 in Multimedia Appendix 1). For PM_10_, 8326 (95% eCI 5980-10,690), 7565 (95% eCI 5130-9602), 4487 (95% eCI 3068-5843), 1991 (95% eCI 1360-2582), and 299 (95% eCI 209-384) deaths were caused, with daily concentrations exceeding the WHO targets (45, 50, 75, 100, and 150 μg/m^3^), respectively (Table S8 in Multimedia Appendix 1). Therefore, 5.4%, 5.7%, and 5.7% of all deaths in Guangzhou were preventable by reaching the lowest target levels of PM_1_, PM_2.5_, and PM_10_, respectively_._

**Table 3:** The number of deaths from cause-specific disease (95% empirical CI) advanced by particulate matters (PM) with an aerodynamic diameter less than 1 µm (PM_1_) with the concentrations exceeding the target levels from 2014 to 2016.

Cause of death	PM_1_ targets (μg/m^3^)				
	10	20	30	40	55
All cause	7921 (4454 to 11,206)	6790 (3704 to 9611)	4899 (2696 to 6971)	3053 (1790 to 4393)	1278 (711 to 1831)
Nonaccidental	7717 (4206 to 11,288)	6616 (3672 to 9204)	4776 (2592 to 6768)	2977 (1617 to 4230)	1246 (714 to 1719)
Circulatory disease	4263 (2374 to 6097)	3682 (1998 to 5241)	2688 (1455 to 3861)	1704 (970 to 2372)	727 (428 to 1057)
Chronic rheumatic heart diseases	2659 (1481 to 3744)	2292 (1300 to 3199)	1669 (1014 to 2367)	1060 (588 to 1497)	444 (245 to 609)
Hypertensive diseases	547 (–43 to 1020)	474 (4 to 858)	346 (11 to 625)	221 (17 to 412)	97 (–2 to 178)
Ischemic heart disease	886 (–402 to 2048)	767 (–284 to 1836)	561 (–166 to 1271)	355 (–182 to 879)	152 (–51 to 375)
Acute ischemic heart disease	185 (–688 to 962)	160 (–612 to 887)	118 (–457 to 619)	76 (–308 to 400)	33 (–135 to 184)
Acute myocardial infarction	257 (–595 to 1033)	223 (–550 to 896)	164 (–415 to 666)	104 (–249 to 429)	45 (–116 to 178)
Myocardial infarction	255 (–549 to 1051)	221 (–439 to 894)	163 (–410 to 661)	104 (–218 to 406)	45 (–104 to 180)
Chronic ischemic heart disease	691 (–218 to 1501)	596 (–147 to 1262)	433 (–111 to 944)	271 (–56 to 586)	115 (–43 to 247)
Other forms of heart disease	–125 (–581 to 229)	–107 (–473 to 178)	–76 (–363 to 137)	–46 (–224 to 79)	–19 (–89 to 35)
Cerebrovascular	2592 (1426 to 3681)	2234 (1287 to 3179)	1628 (862 to 2271)	1035 (553 to 1422)	434 (246 to 597)
Stroke	1320 (359 to 2125)	1140 (369 to 1858)	836 (318 to 1333)	532 (150 to 848)	222 (63 to 367)
Intracerebral hemorrhagic stroke	474 (–44 to 948)	412 (–53 to 809)	305 (–60 to 597)	192 (–27 to 382)	81 (–10 to 161)
Ischemic stroke	651 (55 to 1148)	558 (39 to 1003)	405 (31 to 722)	256 (32 to 446)	104 (11 to 187)
Arteries, arterioles, and capillaries	157 (–49 to 293)	135 (–31 to 254)	96 (–27 to 182)	61 (–14 to 110)	26 (–7 to 49)
Respiratory disease	1506 (366 to 2603)	1295 (316 to 2231)	942 (261 to 1585)	600 (161 to 1014)	261 (68 to 438)
Influenza and pneumonia	840 (73 to 1572)	724 (77 to 1344)	527 (15 to 974)	335 (38 to 616)	147 (9 to 262)
Chronic lower respiratory disease	618 (–199 to 1370)	531 (–105 to 1137)	386 (–121 to 815)	246 (–90 to 534)	105 (–40 to 225)
Chronic obstructive pulmonary disease	487 (–258 to 1168)	419 (–257 to 991)	305 (–209 to 729)	195 (–98 to 466)	83 (–48 to 195)
Other respiratory diseases	–4 (–417 to 346)	–3 (–390 to 315)	–3 (–293 to 232)	–2 (–201 to 156)	–1 (–90 to 73)
Digestive disease	–482(–1132 to 46)	–415 (–923 to 8)	–299 (–704 to 15)	–183 (–434 to 27)	–75 (–176 to 8)
Esophagus, stomach, and duodenum	68 (–214 to 270)	59 (–173 to 238)	44 (–156 to 167)	28 (–76 to 109)	12 (–35 to 45)
Liver	–274 (–706 to 58)	–234 (–598 to 59)	–166 (–430 to 34)	–101 (–262 to 17)	–46 (–118 to 8)
Other digestive diseases	–231 (–613 to 25)	–202 (–538 to 29)	–147 (–376 to 6)	–90 (–226 to 13)	–33 (–88 to 4)
Nervous disease	201 (–52 to 398)	172 (–48 to 332)	124 (–45 to 247)	78 (–29 to 154)	33 (–11 to 63)
Genitourinary disease	–241 (–650 to 102)	–208 (–569 to 115)	–152 (–430 to 72)	–95 (–269 to 46)	–41(–113 to 19)
Urinary disease	–241 (–721 to 94)	–208 (–591 to 74)	–152 (–423 to 72)	–95 (–263 to 47)	–41 (–112 to 14)
Renal failure	–171 (–509 to 67)	–146 (–453 to 74)	–106 (–359 to 47)	–67 (–217 to 24)	–29 (–96 to 16)
External causes	206 (–560 to 865)	177 (–424 to 736)	127 (–282 to 546)	78 (–225 to 314)	33 (–81 to 136)
Road traffic injury	–176 (–616 to 118)	–151 (–490 to 93)	–109 (–348 to 65)	–67 (–226 to 46)	–28 (–98 to 18)
Intentional self-harm	67 (–202 to 252)	57 (–153 to 221)	40 (–116 to 160)	25 (–84 to 96)	10 (–33 to 39)
Endocrine diseases	162 (–420 to 707)	140 (–415 to 620)	102 (–278 to 438)	64 (–186 to 273)	28 (–78 to 111)
Diabetes	201 (–312 to 608)	174 (–264 to 526)	128 (–163 to 353)	82 (–118 to 253)	37 (–46 to 105)
Neoplasms	1724 (128 to 3348)	1458 (223 to 2676)	1029 (44 to 1947)	620 (86 to 1144)	247 (13 to 462)
Pancreas	17 (–275 to 254)	14 (–289 to 212)	10 (–186 to 153)	6 (–102 to 87)	2 (–37 to 31)

### Sensitivity Analyses

In the sensitivity analyses, when we used 3-6 dfs for relative humidity, mean temperature, and air pressure and spline function with 5-9 dfs per year for calendar days, the effect sizes remained stable. In the two-pollutant models, after separately including ozone, sulfur dioxide, and carbon monoxide in the main model, the effect estimations of PMs on mortality remained similar and statistically significant (Figures S5-S7 in Multimedia Appendix 1).

## Discussion

### Principal Findings

To the best of our knowledge, this is the first investigation to assess the associations between size-fractionated PM exposure and deaths from a wide range of causes within the same population. In comparison to PM_10_ and PM_2.5_, we observed a stronger association between PM_1_ and mortality risk from cardiorespiratory diseases and neoplasms. Among more specific diseases, significant effect estimates of PM_1_ were found among deaths due to hypertensive diseases, chronic rheumatic heart diseases, stroke (notably ischemic stroke), influenza, and pneumonia. Over 5% of all deaths were caused by PMs_,_ with the daily concentrations exceeding the target levels in the WHO’s air quality guidelines.

### Comparison With Prior Work

Consistent with previous research [8,26-28], we found that the health risk increased with the shrinkage in PM size, with PM_1_ ranking the highest in effect estimates. For instance, Zhang et al [8] revealed that for every 10 μg/m^3^ increment in PM_1_, PM_2.5_, and PM_10_, the hospital admissions due to cardiovascular diseases increased by 6.7%, 4.5%, and 3.4%, respectively. This gradient in health risk might be mainly attributed to the different particle sizes of PMs, which affect their deposition and absorption in the lungs [7,29]. As the particle size decreases, the surface area increases, leading to a greater catalytic effect on the generation of active oxygen. Smaller molecular size is also conducive to cell absorption, which may further cause the production of high reactive oxygen species, DNA damage, and an increase in interleukin 8 [30]. It is important to note that the relationships between size-fractionated PMs and cause-specific mortality were mostly monotonical in our study, suggesting no safe threshold for these PM pollutants and that any efforts to reduce the PM levels could achieve appreciable health benefits.

A large number of diseases were suscepitable to size-fractionated PMs in our study, most of them originating from cardiorespiratory systems. Previous studies mainly focused on the broad categories of diseases and found similar results—the mortality risk was highest for circulatory and respiratory diseases in general [9]. For the subcategories of causes of deaths, significant impact was detected among deaths due to hypertensive diseases, chronic rheumatic heart diseases, stroke (notably ischemic stroke), influenza, and pneumonia for all size-fractionated PMs. This highlights the importance of prioritizing resource allocations during exposure periods and raising awareness of self-protection among patients with these diseases.

It is interesting to note the positive association between PMs and cancer mortality. Attribution bias might partially explain this finding, that is, the incorrect recording of cause of death as cancer, rather than recording the actual causes, if there is a diagnosis of cancer in a person’s medical history. In other words, the risk of dying from comorbidities (eg, cardiorespiratory diseases and infection) in patients with cancer increases during highly polluted days, but the cause of death may be falsely assigned to cancer. This bias is almost inevitable, even in developed countries, where death registration data are fairly good [31]. Meanwhile, we cannot exclude the possibility that there is a true relationship between short-term exposure to PMs and cancer mortality. Indeed, there are studies showing the acute impact of PMs on cancer hospitalization and mortality [32,33]. However, we acknowledge that the underlying mechanism remains poorly characterized. Although it is unknown whether this elevated risk of death among patients with cancer is due to competing mortality or cancer-specific mortality, the message is clear—patients with cancer could be vulnerable when exposed to air pollution, and significant efforts are needed to raise awareness among these populations and provide the much-needed protective measures.

Furthermore, the association of size-fractionated PMs with mortality varied by season, age group, and gender. The mortality risks of PMs were stronger during warm seasons, which is in line with previous findings [6,9]. The sources of PMs varied at different temperature levels, and PM_1_ may be present in more toxic forms in warmer seasons, potentially transmiting faster and staying longer in the air [34]. During warm seasons, people are prone to open windows and go outdoors more frequently [35], and the absorption of air pollutants may also be enhanced through the temperature regulation system (eg, by increasing sweating, minute ventilation, and cardiac output) [34]. People aged 85 years and older were the most vulnerable to PMs. With the rapid aging in Guangzhou, the death burden is set to increase if stricter controls on air pollution are unavailable in the coming years. The gender effect varies by different health outcomes. For instance, Hu et al [6] (2018) and Yin et al [9] (2020) found that the effect of PM_1_ on all-cause mortality among female individuals was larger than that in their male counterparts. Yin et al [27] (2020) reported a stronger impact of PM_1_ on male individuals for circulatory diseases. The varying impact of PMs between genders may be partly explained by the biological differences between male and female individuals, such as different gas-blood barrier permeability, particle deposition efficiency, and hormonal status [27]. Another reason may be due to between-gender demographic and behavioral differences, including occupational type, smoking, and lifestyle, which may modify the PM-mortality relationship [6].

### Limitations

Some limitations of this study need to be noted. First, there might be diagnostic errors, and therefore, misclassification in the specific causes of death. However, the influence is likely to be small in our study because several quality control measures are available in the Guangzhou death registration system to ensure the accuracy of death classifications [36]. Second, we estimated the death burden of size-fractionated PMs by using several recommended target levels as the reference. However, since no safe threshold was detected for all PMs, our calculations are largely conservative in terms of the health benefits that can be achieved by reducing PMs levels. Finally, as an ecological study, we were unable to explore the individual-level PM-mortality association or control for potential covariates at an individual level, such as smoking habits and occupational exposures. Therefore, the causal relationship cannot be tested in our study. Our study was mainly intended to generate hypotheses on their association, and further research is warranted to test and validate these findings.

### Conclusions

Our study indicates that smaller particles are more hazardous to human health. Among specific diseases, significant effect estimates of PM_1_ were found for deaths due to hypertensive diseases, chronic rheumatic heart diseases, stroke, influenza, and pneumonia. Our findings highlight the importance of size-based strategies in the control of PMs and management of their health impact.

## Appendix

Multimedia Appendix 1 Additional statistics.

## Abbreviations

eCI: empirical confidence interval
ILI: influenza-like illness
PM: particulate matter
WHO: World Health Organization

## Data Availability

The data sets generated and analyzed during this study are available from the corresponding author on reasonable request and with the permission of Guangzhou Center for Disease Control and Prevention.
