# Supplementary material for: Mortality Risk and Burden From a Spectrum of Causes in Relation to Size-Fractionated Particulate Matters: Time Series Analysis
Source: JMIR Public Health Surveill. 2023 Oct 9;9:e41862. doi: 10.2196/41862 (PMC10637369; doi:10.2196/41862)
Supplement: Multimedia Appendix 1 [file publichealth_v9i1e41862_app1.docx]

**Supplementary materials**

Mortality risk and burden from a spectrum of causes in relation to size-fractionated particulate matters: A time-series analysis

**Table of contents**

| **Title** |  | **Page** |
| --- | --- | --- |
| Table S1. Summary statistics of cause-specific mortality. |  | 3 |
| Table S2. The PM_2.5_ and PM_1_ targets in this study. |  | 4 |
| Table S3. Correlations (Spearman correlation coefficient) between air pollution and weather conditions |  | 5 |
| Table S4. Percentage change (95%CI) in cause-specific mortality with per 10 μg/m^3^ increase in PM_1_, PM_2.5_ and PM_10_ at 0-3 days in Guangzhou, China. |  | 6 |
| Table S5. Percentage change (95%CI) in mortality with per 10 μg/m^3^ increase in PM_2.5_ at lag 0-3 days in Guangzhou, China, stratified by age group and gender. |  | 7 |
| Table S6. Percentage change (95%CI) in mortality with per 10 μg/m^3^ increase in PM_10_ at lag 0-3 days in Guangzhou, China, stratified by age group and gender. |  | 8 |
| Table S7. The cause-specific mortality advanced by PM_2.5_ if the pollution levels exceeded the standard targets during 2014-2016. |  | 9 |
| Table S8. The cause-specific mortality advanced by PM_10_ if the pollution levels exceeded the standard targets during 2014-2016. |  | 10 |
| Fig. S1 The location of Guangzhou, China. |  | 11 |
| Fig. S2 The concentration-response curves of particulate matter and cause-specific mortality at lag 0-3 day in Guangzhou, China, using five degrees of freedom in smoothness of particulate matter. |  | 12 |
| Fig. S3 The concentration-response curves of particulate matter and cause-specific mortality at lag 0-3 day in Guangzhou, China, using seven degrees of freedom in smoothness of particulate matter. |  | 13 |
| Fig. S4 The concentration-response curves of particulate matter and cause-specific mortality at lag 0-3 day in Guangzhou, China, using nine degrees of freedom in smoothness of particulate matter. |  | 14 |
| Fig. S5 Sensitivity analyses for cumulative relative risk (95%CI) of mortality associated with a 10 μg/m^3^ increase in PM_1_ at lag 0-3 day. |  | 15 |
| Fig. S6 Sensitivity analyses for cumulative relative risk (95%CI) of mortality associated with a 10 μg/m^3^ increase in PM_2.5_ at lag 0-3 day. |  | 16 |
| Fig. S7 Sensitivity analyses for cumulative relative risk (95%CI) of mortality associated with a 10 μg/m^3^ increase in PM_10_ at lag 0-3 day. |  | 17 |

Table S1. Summary statistics for cause-specific mortality.

| Diseases | ICD-10 | Total | Mean(range) |
| --- | --- | --- | --- |
| All-cause | A00-Z99 | 146459 | 134 (86, 251) |
| Non-accidental | A00-R99 | 138396 | 126 (80, 238) |
| Circulatory disease | I00-I99 | 56587 | 52 (21, 115) |
| Chronic rheumatic heart diseases | I05-I09 | 24651 | 22 (6, 50) |
| Hypertensive diseases | I10-I15 | 5347 | 5 (0, 17) |
| Ischemic heart disease | I20-I25 | 23851 | 22 (6, 48) |
| Acute ischemic heart disease | I20-I22, I24 | 11374 | 10 (1, 25) |
| Acute myocardial infarction | I21-I22 | 10696 | 10 (1, 25) |
| Myocardial infarction | I21-I23 | 10699 | 10 (1, 25) |
| Chronic ischemic heart disease | I25 | 12474 | 11 (1, 30) |
| Other forms of heart disease | I30-I52 | 1984 | 2 (0, 7) |
| Cerebrovascular | I60-I69 | 23469 | 21 (6, 49) |
| Stroke | I60-I64 | 14793 | 13 (0, 29) |
| Intracerebral hemorrhagic stroke | I61 | 5257 | 5 (0, 14) |
| Ischemic stroke | I63 | 6051 | 6 (0, 17) |
| Arteries, arterioles and capillaries | I70-I79 | 791 | 1 (0, 5) |
| Respiratory disease | J00-J99 | 21395 | 20 (6, 47) |
| Influenza and pneumonia | J09-J18 | 10883 | 10 (1, 29) |
| Chronic lower respiratory | J40-J47 | 10074 | 9 (1, 22) |
| Chronic obstructive pulmonary disease | J40-J44 | 9443 | 9 (0, 22) |
| Other respiratory | J95-J99 | 2349 | 2 (0, 11) |
| Digestive disease | K00-K93 | 4587 | 4 (0, 12) |
| Oesophagus, stomach and duodenum | K20-K31 | 1061 | 1 (0, 5) |
| Liver | K70-K77 | 1427 | 1 (0, 7) |
| Other digestive | K90-K93 | 937 | 1 (0, 5) |
| Nervous disease | G00-G99 | 1303 | 1 (0, 7) |
| Genitourinary disease | N00-N99 | 1930 | 2 (0, 8) |
| Urinary | N00-N39 | 1930 | 2 (0, 8) |
| Renal failure | N17-N19 | 978 | 1 (0, 5) |
| External causes | V01-Y89 | 8052 | 7 (0, 17) |
| Road traffic injury | V01-V89 | 1291 | 1 (0, 6) |
| Intentional self-harm | X60-X84 | 1010 | 1 (0, 5) |
| Endocrine diseases | D50-D89, E00-E90 | 5469 | 5 (0, 15) |
| Diabetes | E10-E14 | 3477 | 3 (0, 12) |
| Neoplasms | C00-D48 | 41709 | 38 (17, 58) |
| Pancreas | C25 | 1232 | 1 (0, 6) |

Table S2. The PM_2.5_ and PM_1_ targets in this study.

| Level |  |  | PM_2.5_ target | |  | PM_1_ target |
| --- | --- | --- | --- | --- | --- | --- |
|  |  |  | Concentration (μg/m^3^) | Percentile (%) |  | Concentration (μg/m^3^) |
| AQG |  |  | 15 | 6 |  | 10 |
| IT4 |  |  | 25 | 31 |  | 20 |
| IT3 |  |  | 37.5 | 58 |  | 30 |
| IT2 |  |  | 50 | 78 |  | 40 |
| IT1 |  |  | 75 | 94 |  | 55 |

Note. AQG represents the WHO’s new air quality guideline. IT1-IT4 denote the interim targets 1 to 4.

Table S3. Correlations (Spearman correlation coefficient) between air pollution and weather conditions

| Variables | PM_1_ | PM_2.5_ | PM_10_ | O_3_ | SO_2_ | NO_2_ | CO | Mean temperature | Relative humidity | Air pressure |
| --- | --- | --- | --- | --- | --- | --- | --- | --- | --- | --- |
| PM_1_ |  |  |  |  |  |  |  |  |  |  |
| PM_2.5_ | 0.85^**^ |  |  |  |  |  |  |  |  |  |
| PM_10_ | 0.89^**^ | 0.96^**^ |  |  |  |  |  |  |  |  |
| O_3_ | 0.15^**^ | 0.28^**^ | 0.35^**^ |  |  |  |  |  |  |  |
| SO_2_ | 0.58^**^ | 0.69^**^ | 0.72^**^ | 0.39^**^ |  |  |  |  |  |  |
| NO_2_ | 0.73^**^ | 0.77^**^ | 0.76^**^ | 0.01 | 0.59^**^ |  |  |  |  |  |
| CO | 0.55^**^ | 0.60^**^ | 0.53^**^ | -0.19^**^ | 0.31^**^ | 0.65^**^ |  |  |  |  |
| Mean temperature | -0.46^**^ | -0.32^**^ | -0.26^**^ | 0.48^**^ | 0.03 | -0.39^**^ | -0.45^**^ |  |  |  |
| Relative humidity | -0.21^**^ | -0.25^**^ | -0.32^**^ | -0.48^**^ | -0.24^**^ | 0.07^**^ | 0.15^**^ | 0.11^**^ |  |  |
| Air pressure | 0.47^**^ | 0.36^**^ | 0.34^**^ | -0.28^**^ | 0.06^*^ | 0.35^**^ | 0.37^**^ | -0.88^**^ | -0.32^**^ |  |

Note: *: P<0.05; **: P<0.01.

Table S4. Percentage change (95%CI) in cause-specific mortality with per 10 μg/m^3^ increase in PM_1_, PM_2.5_ and PM_10_ at 0-3 days in Guangzhou, China.

| Variables | PM_1_ | PM_2.5_ | PM_10_ |
| --- | --- | --- | --- |
| **All-cause** | 2.00(1.08,2.92) | 1.54(0.93,2.16) | 1.38(0.95,1.82) |
| **Non-accidental** | 2.06(1.13,3.00) | 1.59(0.96,2.22) | 1.41(0.96,1.86) |
| **Circulatory disease** | 2.78(1.46,4.13) | 2.25(1.37,3.15) | 1.85(1.21,2.49) |
| Chronic rheumatic heart diseases | 4.11(2.24,6.02) | 2.99(1.74,4.26) | 2.37(1.48,3.27) |
| Hypertensive diseases | 3.85(-0.04,7.88) | 4.05(1.40,6.76) | 3.30(1.40,5.22) |
| Ischemic heart disease | 1.33(-0.57,3.26) | 1.30(0.01,2.60) | 1.09(0.17,2.02) |
| Acute ischemic heart disease | 0.57(-2.03,3.23) | 0.49(-1.28,2.28) | 0.61(-0.66,1.90) |
| Acute myocardial infarction | 0.84(-1.83,3.59) | 0.55(-1.26,2.40) | 0.69(-0.62,2.02) |
| Myocardial infarction | 0.84(-1.84,3.59) | 0.55(-1.27,2.40) | 0.69(-0.63,2.02) |
| Chronic ischemic heart disease | 2.03(-0.51,4.64) | 2.04(0.32,3.78) | 1.53(0.30,2.77) |
| Other forms of heart disease | -2.16(-8.33,4.44) | -2.03(-6.29,2.42) | -0.96(-4.04,2.21) |
| Cerebrovascular | 4.21(2.29,6.17) | 3.07(1.78,4.37) | 2.43(1.52,3.35) |
| Stroke | 3.32(0.97,5.73) | 2.52(0.94,4.12) | 1.92(0.79,3.06) |
| Intracerebral hemorrhagic stroke | 3.33(-0.41,7.21) | 2.77(0.24,5.36) | 2.06(0.25,3.90) |
| Ischemic stroke | 4.13(0.39,8.01) | 3.18(0.66,5.76) | 2.42(0.63,4.24) |
| Arteries, arterioles and capillaries | 8.45(-1.74,19.71) | 5.27(-1.44,12.42) | 3.70(-1.12,8.75) |
| **Respiratory disease** | 2.61(0.63,4.63) | 2.53(1.18,3.91) | 2.21(1.24,3.19) |
| Influenza and pneumonia | 2.87(0.17,5.64) | 2.72(0.87,4.60) | 2.34(1.01,3.68) |
| Chronic lower respiratory | 2.27(-0.59,5.21) | 2.31(0.35,4.32) | 2.14(0.72,3.57) |
| Chronic obstructive pulmonary disease | 1.88(-1.07,4.92) | 2.17(0.14,4.25) | 2.13(0.66,3.61) |
| Other respiratory | -0.05(-5.35,5.53) | 0.09(-3.58,3.89) | 0.19(-2.49,2.94) |
| **Digestive disease** | -3.46(-7.16,0.39) | -2.58(-5.15,0.06) | -1.27(-3.14,0.64) |
| Oesophagus, stomach and duodenum | 2.34(-5.85,11.25) | -2.34(-7.76,3.41) | -1.68(-5.70,2.50) |
| Liver | -6.05(-12.82,1.25) | -3.93(-8.72,1.11) | -1.95(-5.48,1.71) |
| Other digestive | -7.30(-15.10,1.23) | -5.10(-10.64,0.79) | -2.71(-6.77,1.53) |
| **Nervous disease** | 6.24(-1.43,14.50) | 4.73(-0.48,10.21) | 2.58(-1.14,6.43) |
| **Genitourinary disease** | -4.03(-9.76,2.07) | -2.06(-6.10,2.16) | -0.26(-3.21,2.78) |
| Urinary | -4.03(-9.76,2.07) | -2.06(-6.10,2.16) | -0.26(-3.21,2.78) |
| Renal failure | -5.44(-13.39,3.24) | -1.23(-7.00,4.89) | 0.60(-3.62,5.00) |
| **External causes** | 0.93(-2.27,4.23) | 0.72(-1.45,2.94) | 0.95(-0.63,2.55) |
| Road traffic injury | -4.39(-11.73,3.56) | -0.94(-6.13,4.54) | 0.02(-3.78,3.97) |
| Intentional self-harm | 2.52(-6.12,11.96) | 1.74(-4.28,8.13) | 1.34(-3.04,5.91) |
| **Endocrine diseases** | 1.05(-2.69,4.95) | 1.28(-1.27,3.91) | 1.10(-0.74,2.98) |
| Diabetes | 2.09(-2.54,6.94) | 2.16(-1.01,5.42) | 2.06(-0.23,4.41) |
| **Neoplasms** | 1.55(0.11,3.01) | 0.69(-0.28,1.67) | 0.71(0.01,1.40) |
| Pancreas | 0.50(-7.44,9.13) | 0.12(-5.23,5.76) | -0.16(-4.02,3.86) |

Table S5. Percentage change (95%CI) in mortality with per 10 μg/m^3^ increase in PM_2.5_ at lag 0-3 days in Guangzhou, China, stratified by age group and gender.

| Variables | All-cause | Non-accidental | Cardiovascular | Respiratory | Neoplasms |
| --- | --- | --- | --- | --- | --- |
| Total | 1.54(0.93,2.16) | 1.59(0.96,2.22) | 2.25(1.37,3.15) | 2.53(1.18,3.91) | 0.69(-0.28,1.67) |
| Season |  |  |  |  |  |
| Cold | 1.46(0.78,2.15) | 1.53(0.84,2.24) | 2.02(1.05,3.01) | 2.66(1.16,4.18) | 0.54(-0.56,1.65) |
| Hot | 1.84(0.59,3.10) | 1.79(0.52,3.08) | 3.20(1.31,5.12) | 2.08(-0.68,4.91) | 1.15(-0.70,3.03) |
| Age, years |  |  |  |  |  |
| 0-64 | 0.94(-0.05,1.94) | 1.04(-0.02,2.10) | 1.62(-0.43,3.71) | 5.03(0.32,9.95) | 0.13(-1.27,1.55) |
| 65-74 | 0.97(-0.27,2.21) | 0.91(-0.34,2.18) | 1.06(-0.94,3.09) | -0.06(-3.78,3.81) | 1.22(-0.69,3.18) |
| 75-84 | 1.66(0.69,2.64) | 1.72(0.73,2.72) | 2.39(1.03,3.76) | 2.24(0.15,4.38) | 0.41(-1.37,2.21) |
| 85+ | 2.35(1.26,3.44) | 2.38(1.28,3.48) | 2.93(1.44,4.45) | 3.06(1.05,5.12) | 2.78(-0.37,6.04) |
| Gender |  |  |  |  |  |
| Male | 1.37(0.65,2.10) | 1.41(0.67,2.16) | 2.62(1.49,3.76) | 2.87(1.13,4.64) | 0.28(-0.90,1.48) |
| Female | 1.77(0.92,2.63) | 1.83(0.97,2.70) | 1.88(0.66,3.12) | 2.07(0.09,4.08) | 1.41(-0.21,3.05) |

Table S6. Percentage change (95%CI) in mortality with per 10 μg/m^3^ increase in PM_10_ at lag 0-3 days in Guangzhou, China, stratified by age group and gender.

| Variables | All-cause | Non-accidental | Cardiovascular | Respiratory | Neoplasms |
| --- | --- | --- | --- | --- | --- |
| Total | 1.38(0.95,1.82) | 1.41(0.96,1.86) | 1.85(1.21,2.49) | 2.21(1.24,3.19) | 0.71(0.01,1.40) |
| Season |  |  |  |  |  |
| Cold | 1.28(0.79,1.76) | 1.31(0.82,1.81) | 1.66(0.96,2.36) | 2.24(1.17,3.33) | 0.53(-0.25,1.32) |
| Hot | 1.78(0.89,2.67) | 1.77(0.86,2.68) | 2.62(1.28,3.98) | 2.10(0.11,4.12) | 1.26(-0.08,2.61) |
| Age, years |  |  |  |  |  |
| 0-64 | 0.82(0.11,1.54) | 0.88(0.13,1.64) | 1.28(-0.19,2.77) | 3.88(0.54,7.34) | 0.25(-0.75,1.27) |
| 65-74 | 1.07(0.19,1.95) | 1.03(0.13,1.93) | 1.04(-0.39,2.48) | 0.67(-2.03,3.45) | 1.06(-0.30,2.45) |
| 75-84 | 1.51(0.82,2.21) | 1.56(0.85,2.27) | 1.98(1.01,2.96) | 2.15(0.65,3.68) | 0.52(-0.76,1.81) |
| 85+ | 1.97(1.20,2.75) | 1.96(1.17,2.75) | 2.32(1.26,3.40) | 2.40(0.95,3.87) | 2.44(0.17,4.76) |
| Gender |  |  |  |  |  |
| Male | 1.25(0.73,1.77) | 1.26(0.73,1.79) | 1.91(1.11,2.72) | 2.52(1.27,3.78) | 0.46(-0.39,1.32) |
| Female | 1.57(0.96,2.18) | 1.61(0.99,2.23) | 1.78(0.91,2.66) | 1.78(0.35,3.22) | 1.13(-0.02,2.31) |

Table S7. The cause-specific mortality advanced by PM_2.5_ if the pollution levels exceeded the standard targets during 2014-2016.

|  | PM_2.5_ targets (μg/m^3^) | | | | |
| --- | --- | --- | --- | --- | --- |
| Cause | 15 | 25 | 37.5 | 50 | 75 |
| All-cause | 8303(5063,11248) | 7194(4183,9833) | 5504(3245,7620) | 3717(2242,5171) | 1352(840,1861) |
| Non-accidental | 8083(4994,11282) | 7004(4348,9816) | 5360(3192,7304) | 3620(2209,4971) | 1316(866,1822) |
| Circulatory disease | 4656(2898,6363) | 4050(2486,5397) | 3127(1942,4291) | 2142(1303,2869) | 794(520,1079) |
| Chronic rheumatic heart diseases | 2631(1579,3624) | 2285(1285,3162) | 1762(1101,2403) | 1205(745,1631) | 445(263,598) |
| Hypertensive diseases | 760(274,1157) | 663(239,1036) | 513(195,785) | 358(129,556) | 138(47,214) |
| Ischemic heart disease | 1159(-17,2283) | 1009(-23,2011) | 781(56,1520) | 534(-21,1000) | 197(1,375) |
| Acute ischemic heart disease | 214(-594,928) | 187(-510,836) | 146(-417,593) | 100(-248,441) | 38(-94,164) |
| Acute myocardial infarction | 228(-498,878) | 199(-499,835) | 155(-386,609) | 106(-248,444) | 40(-101,157) |
| Myocardial infarction | 227(-543,918) | 198(-472,830) | 154(-358,621) | 106(-259,422) | 39(-102,160) |
| Chronic ischemic heart disease | 925(154,1627) | 802(140,1468) | 617(120,1082) | 419(68,737) | 153(30,265) |
| Other forms of heart disease | -160(-580,146) | -137(-486,123) | -103(-366,106) | -68(-258,74) | -24(-83,25) |
| Cerebrovascular | 2567(1604,3553) | 2229(1351,3062) | 1720(1016,2320) | 1179(709,1604) | 436(264,608) |
| Stroke | 1355(583,2127) | 1181(501,1846) | 916(349,1432) | 630(257,985) | 232(83,355) |
| Intracerebral hemorrhagic stroke | 530(94,960) | 465(49,840) | 363(27,648) | 251(-4,447) | 94(14,171) |
| Ischemic stroke | 680(187,1154) | 588(106,998) | 451(97,743) | 308(80,521) | 110(31,187) |
| Arteries, arterioles and capillaries | 137(-22,273) | 119(-36,244) | 90(-32,178) | 60(-17,115) | 22(-5,43) |
| Respiratory disease | 1949(913,2846) | 1686(851,2489) | 1289(639,1919) | 883(443,1289) | 335(159,488) |
| Influenza and pneumonia | 1062(342,1749) | 919(316,1492) | 702(261,1128) | 482(154,768) | 184(64,295) |
| Chronic lower respiratory | 840(92,1503) | 726(129,1278) | 556(94,1009) | 380(59,666) | 142(28,251) |
| Chronic obstructive pulmonary disease | 745(99,1392) | 645(22,1179) | 495(67,939) | 340(20,619) | 127(5,233) |
| Other respiratory | 9(-397,328) | 8(-357,282) | 6(-275,252) | 4(-192,166) | 2(-79,67) |
| Digestive disease | -485(-1044,6) | -419(-914,17) | -321(-707,4) | -214(-471,1) | -75(-158,4) |
| Oesophagus, stomach and duodenum | -102(-420,135) | -89(-337,103) | -69(-279,80) | -47(-191,58) | -17(-64,20) |
| Liver | -237(-614,48) | -204(-526,56) | -154(-417,55) | -103(-271,24) | -39(-104,9) |
| Other digestive | -212(-513,38) | -184(-460,18) | -144(-353,22) | -95(-247,11) | -32(-83,4) |
| Nervous disease | 207(-14,394) | 179(-29,329) | 136(-16,246) | 91(-10,172) | 35(-3,64) |
| Genitourinary disease | -162(-556,131) | -141(-444,130) | -108(-349,94) | -73(-239,63) | -26(-86,23) |
| Urinary | -162(-543,147) | -141(-466,129) | -108(-393,99) | -73(-261,70) | -26(-83,24) |
| Renal failure | -48(-317,159) | -41(-287,125) | -32(-226,106) | -22(-160,72) | -8(-55,27) |
| External causes | 217(-413,837) | 188(-443,718) | 143(-325,525) | 97(-171,392) | 35(-71,132) |
| Road traffic injury | -48(-354,185) | -42(-315,169) | -32(-235,126) | -21(-176,88) | -7(-60,31) |
| Intentional self-harm | 63(-171,244) | 54(-148,218) | 41(-133,164) | 27(-82,104) | 9(-27,37) |
| Endocrine diseases | 263(-289,733) | 230(-235,642) | 177(-185,491) | 122(-128,343) | 45(-48,129) |
| Diabetes | 277(-141,638) | 242(-130,531) | 187(-91,428) | 130(-68,291) | 50(-24,116) |
| Neoplasms | 1060(-430,2464) | 914(-406,2138) | 690(-227,1675) | 452(-205,1037) | 158(-54,356) |
| Pancreas | 5(-269,229) | 5(-258,200) | 3(-183,142) | 2(-108,92) | 1(-37,32) |

Table S8. The cause-specific mortality advanced by PM_10_ if the pollution levels exceeded the standard targets during 2014-2016.

|  | PM_10_ targets (μg/m^3^) | | | | |
| --- | --- | --- | --- | --- | --- |
| Cause | 45 | 50 | 75 | 100 | 150 |
| All-cause | 8326(5980,10690) | 7565(5130,9602) | 4487(3068,5843) | 1991(1360,2582) | 299(209,384) |
| Non-accidental | 8009(5426,10299) | 7278(5162,9417) | 4316(3020,5526) | 1917(1332,2469) | 289(202,376) |
| Circulatory disease | 4299(2847,5621) | 3919(2683,5179) | 2355(1544,3116) | 1073(724,1408) | 168(118,222) |
| Chronic rheumatic heart diseases | 2359(1483,3160) | 2151(1385,2815) | 1293(836,1734) | 589(397,789) | 89(56,118) |
| Hypertensive diseases | 694(325,1000) | 633(276,916) | 389(194,576) | 178(81,260) | 28(14,41) |
| Ischemic heart disease | 1103(238,2000) | 1005(159,1814) | 602(69,1054) | 274(40,481) | 44(7,80) |
| Acute ischemic heart disease | 304(-374,878) | 278(-314,788) | 168(-195,512) | 76(-82,222) | 13(-15,40) |
| Acute myocardial infarction | 319(-324,891) | 292(-250,818) | 175(-203,475) | 79(-65,225) | 13(-14,37) |
| Myocardial infarction | 318(-299,860) | 290(-305,845) | 175(-178,476) | 79(-75,215) | 13(-15,38) |
| Chronic ischemic heart disease | 784(161,1345) | 714(132,1258) | 424(70,727) | 194(38,335) | 29(6,50) |
| Other forms of heart disease | -83(-398,183) | -75(-370,148) | -43(-208,83) | -18(-91,36) | -3(-12,5) |
| Cerebrovascular | 2297(1521,3071) | 2094(1334,2811) | 1262(851,1662) | 576(372,763) | 88(59,117) |
| Stroke | 1175(511,1820) | 1073(486,1623) | 652(300,990) | 293(115,449) | 44(19,68) |
| Intracerebral hemorrhagic stroke | 453(68,782) | 415(40,753) | 253(41,440) | 115(16,206) | 17(4,30) |
| Ischemic stroke | 584(173,948) | 531(152,858) | 319(101,523) | 142(42,232) | 20(7,33) |
| Arteries, arterioles and capillaries | 109(-33,219) | 100(-36,203) | 57(-17,109) | 26(-7,50) | 4(-1,8) |
| Respiratory disease | 1881(1156,2607) | 1707(1009,2395) | 1024(566,1409) | 467(266,651) | 76(45,107) |
| Influenza and pneumonia | 1008(449,1500) | 914(401,1349) | 548(242,826) | 250(113,375) | 41(20,63) |
| Chronic lower respiratory | 855(349,1387) | 777(264,1251) | 466(168,719) | 212(80,338) | 34(13,56) |
| Chronic obstructive pulmonary disease | 803(273,1278) | 731(253,1174) | 439(136,710) | 201(68,324) | 32(10,52) |
| Other respiratory | 21(-310,273) | 20(-280,251) | 12(-169,158) | 6(-74,76) | 1(-13,11) |
| Digestive disease | -261(-683,111) | -237(-627,96) | -139(-373,76) | -60(-167,29) | -8(-21,4) |
| Oesophagus, stomach and duodenum | -83(-329,97) | -75(-301,105) | -44(-177,56) | -20(-80,24) | -2(-10,3) |
| Liver | -125(-422,93) | -114(-370,91) | -66(-220,50) | -28(-86,22) | -5(-15,3) |
| Other digestive | -123(-353,58) | -113(-313,54) | -66(-193,28) | -27(-85,12) | -3(-9,1) |
| Nervous disease | 131(-61,288) | 119(-67,255) | 69(-29,151) | 32(-12,68) | 5(-3,10) |
| Genitourinary disease | -22(-302,217) | -20(-266,195) | -12(-174,114) | -5(-73,53) | -1(-10,7) |
| Urinary | -22(-311,215) | -20(-275,190) | -12(-172,111) | -5(-80,52) | -1(-9,7) |
| Renal failure | 25(-176,170) | 23(-168,163) | 13(-102,94) | 6(-45,40) | 1(-5,6) |
| External causes | 316(-260,776) | 287(-169,749) | 170(-131,429) | 74(-45,195) | 11(-7,25) |
| Road traffic injury | 1(-248,193) | 1(-237,186) | 1(-141,110) | 0(-56,43) | 0(-7,6) |
| Intentional self-harm | 54(-151,204) | 48(-129,181) | 28(-81,107) | 12(-35,45) | 1(-4,5) |
| Endocrine diseases | 255(-164,635) | 233(-202,573) | 139(-98,349) | 63(-52,160) | 10(-6,25) |
| Diabetes | 295(-18,583) | 269(-21,535) | 163(-21,328) | 76(-8,148) | 12(-1,23) |
| Neoplasms | 1211(36,2360) | 1095(2,2126) | 635(-2,1230) | 270(-6,541) | 37(1,72) |
| Pancreas | -8(-238,182) | -7(-214,159) | -4(-116,92) | -2(-51,36) | 0(-8,6) |


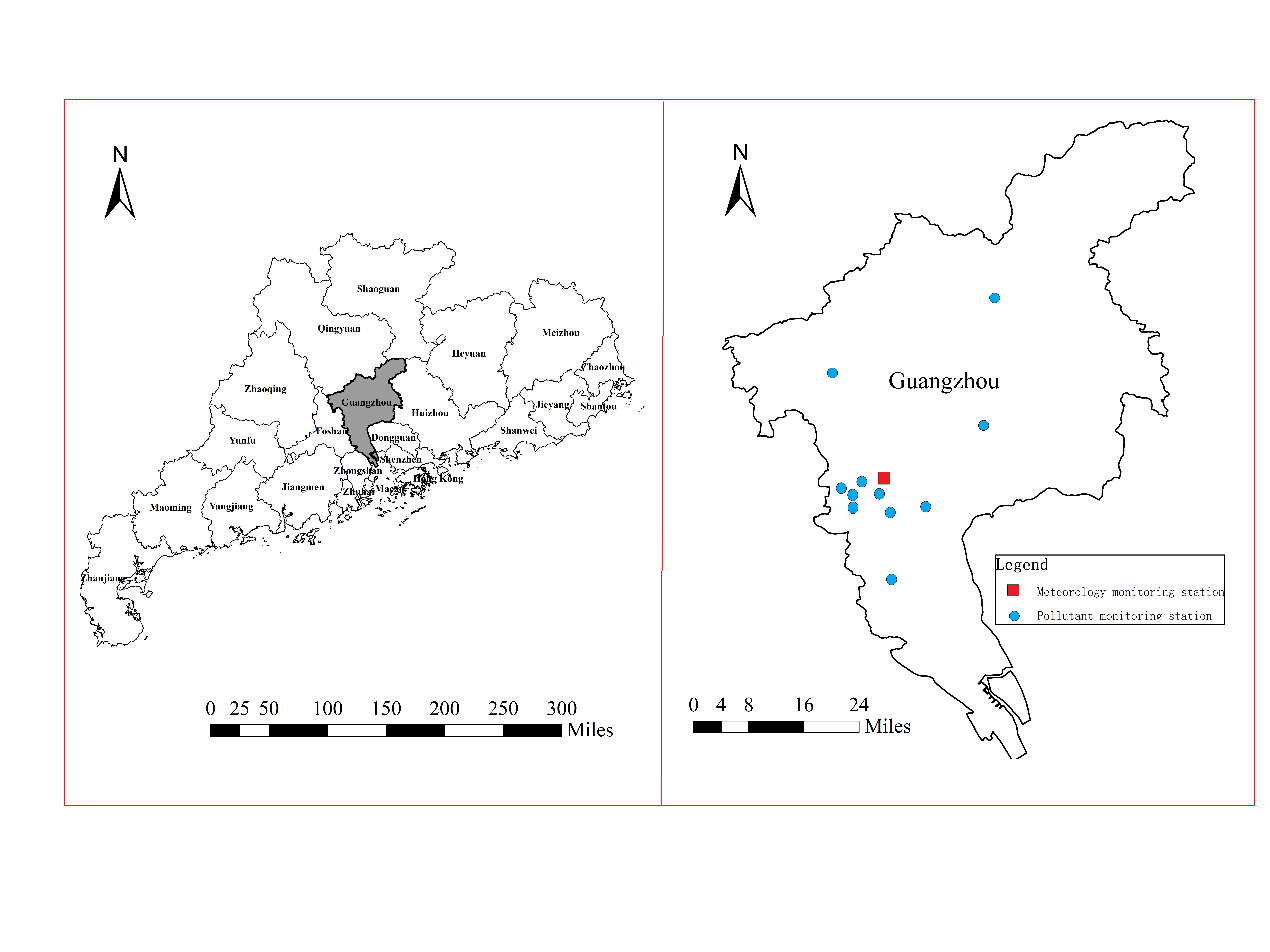


Fig. S1 The location of Guangzhou, China. The left panel shows the location of Guangzhou in Guangdong Province, China; the right panel shows the distributions of 11 air pollution monitoring stations (blue dots) and one weather monitoring station (red square).


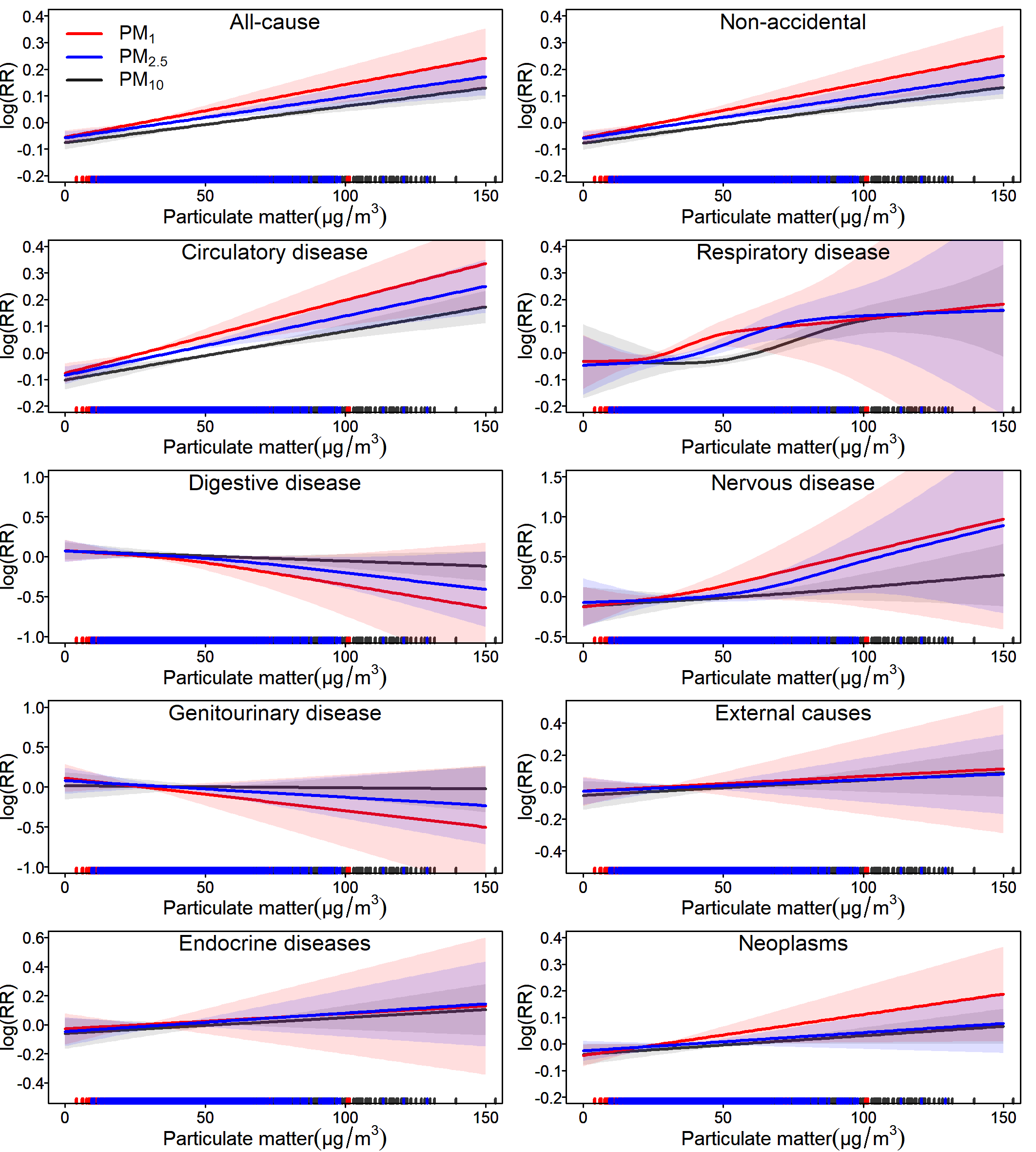


Fig. S2 The concentration-response curves of particulate matter and cause-specific mortality at lag 0-3 day in Guangzhou, China, using five degrees of freedom in smoothness of particulate matter. Red, blue and gray lines respectively denote the PM_1_-mortality association, PM_2.5_-mortality association, and PM_10_-mortality association; the areas denote 95% confidence interval_._


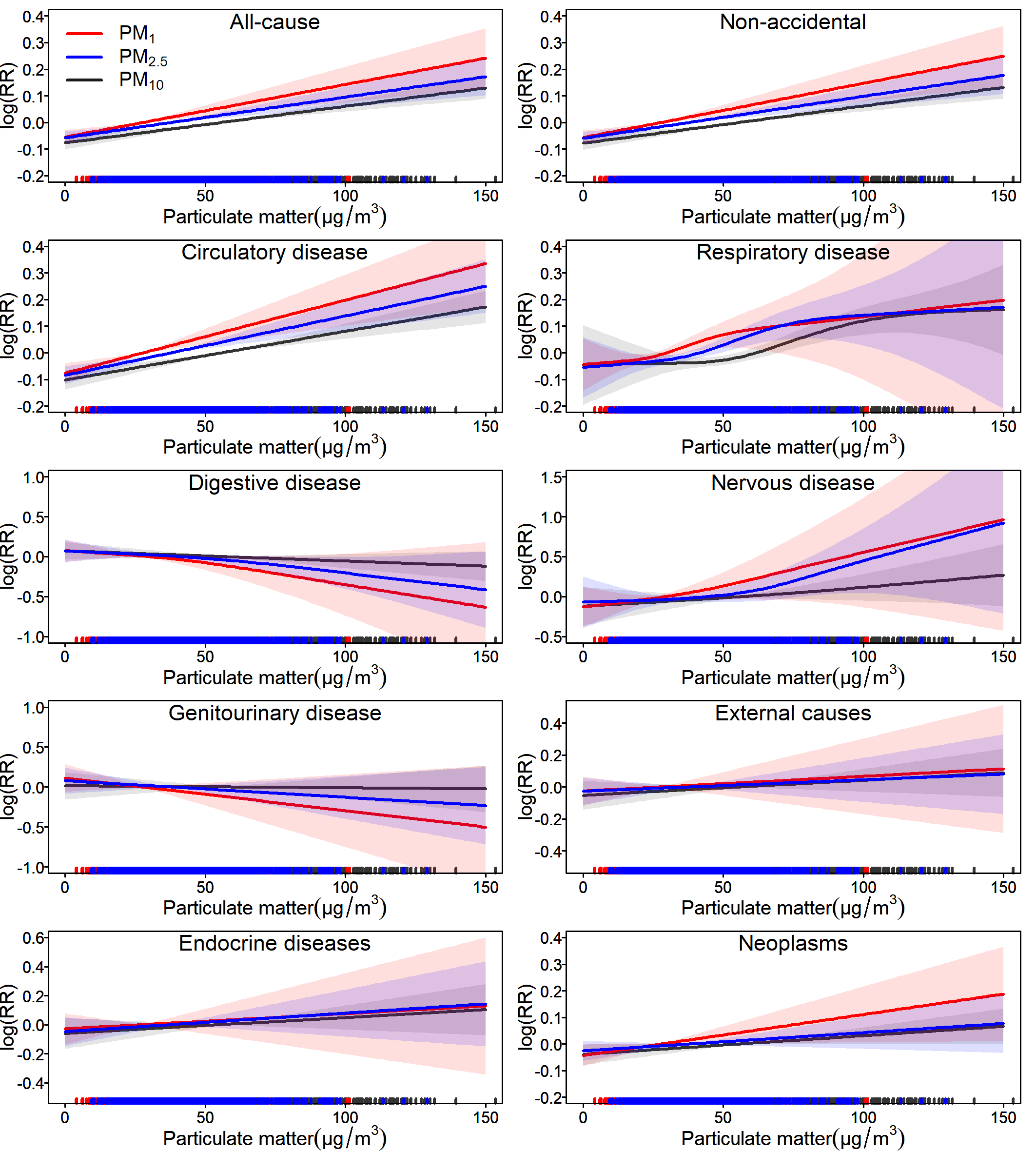


Fig. S3 The concentration-response curves of particulate matter and cause-specific mortality at lag 0-3 day in Guangzhou, China, using seven degrees of freedom in smoothness of particulate matter. Red, blue and gray lines respectively denote the PM_1_-mortality association, PM_2.5_-mortality association, and PM_10_-mortality association; the areas denote 95% confidence interval_._


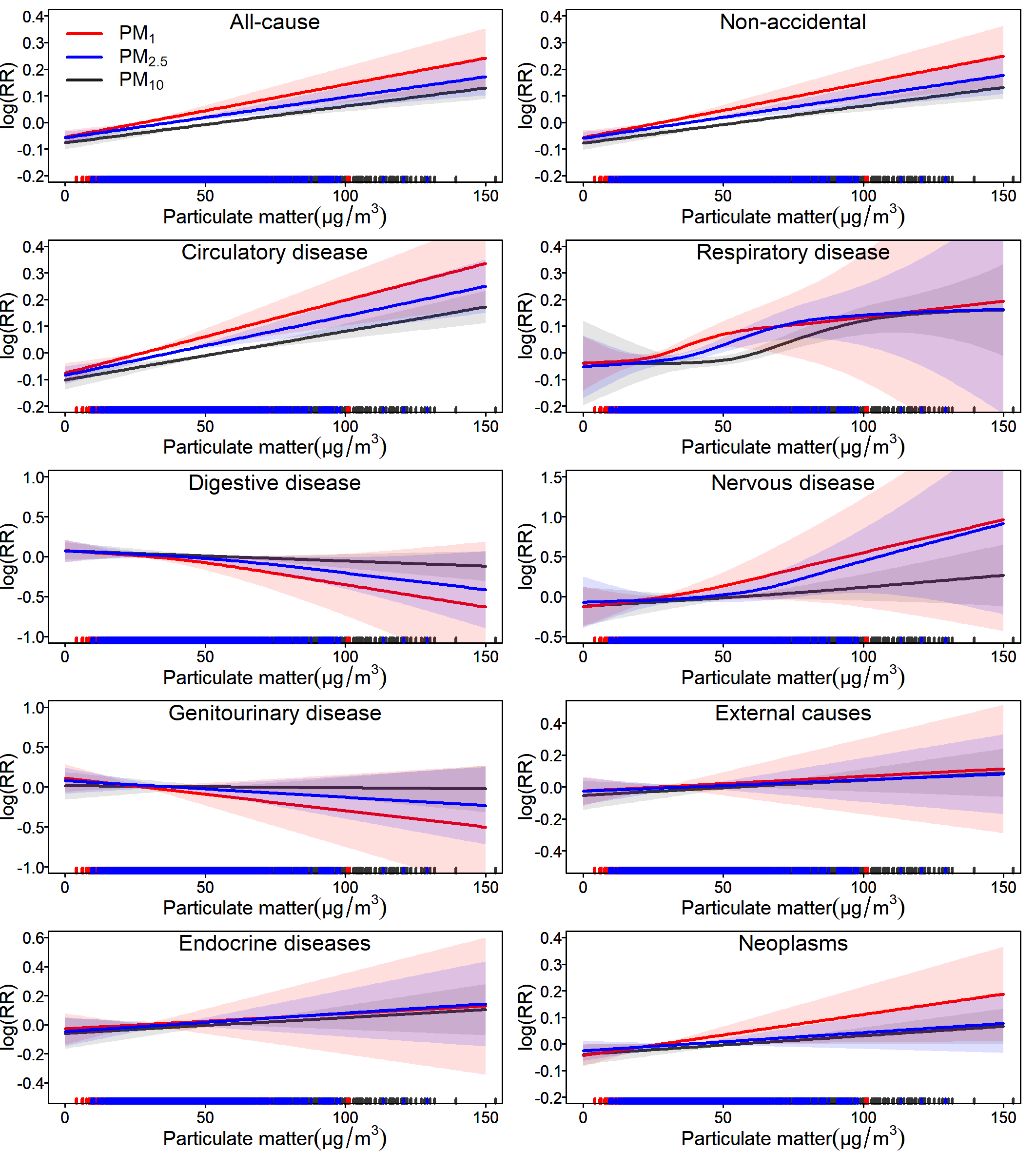


Fig. S4 The concentration-response curves of particulate matter and cause-specific mortality at lag 0-3 day in Guangzhou, China, using nine degrees of freedom in smoothness of particulate matter. Red, blue and gray lines respectively denote the PM_1_-mortality association, PM_2.5_-mortality association, and PM_10_-mortality association; the areas denote 95% confidence interval_._


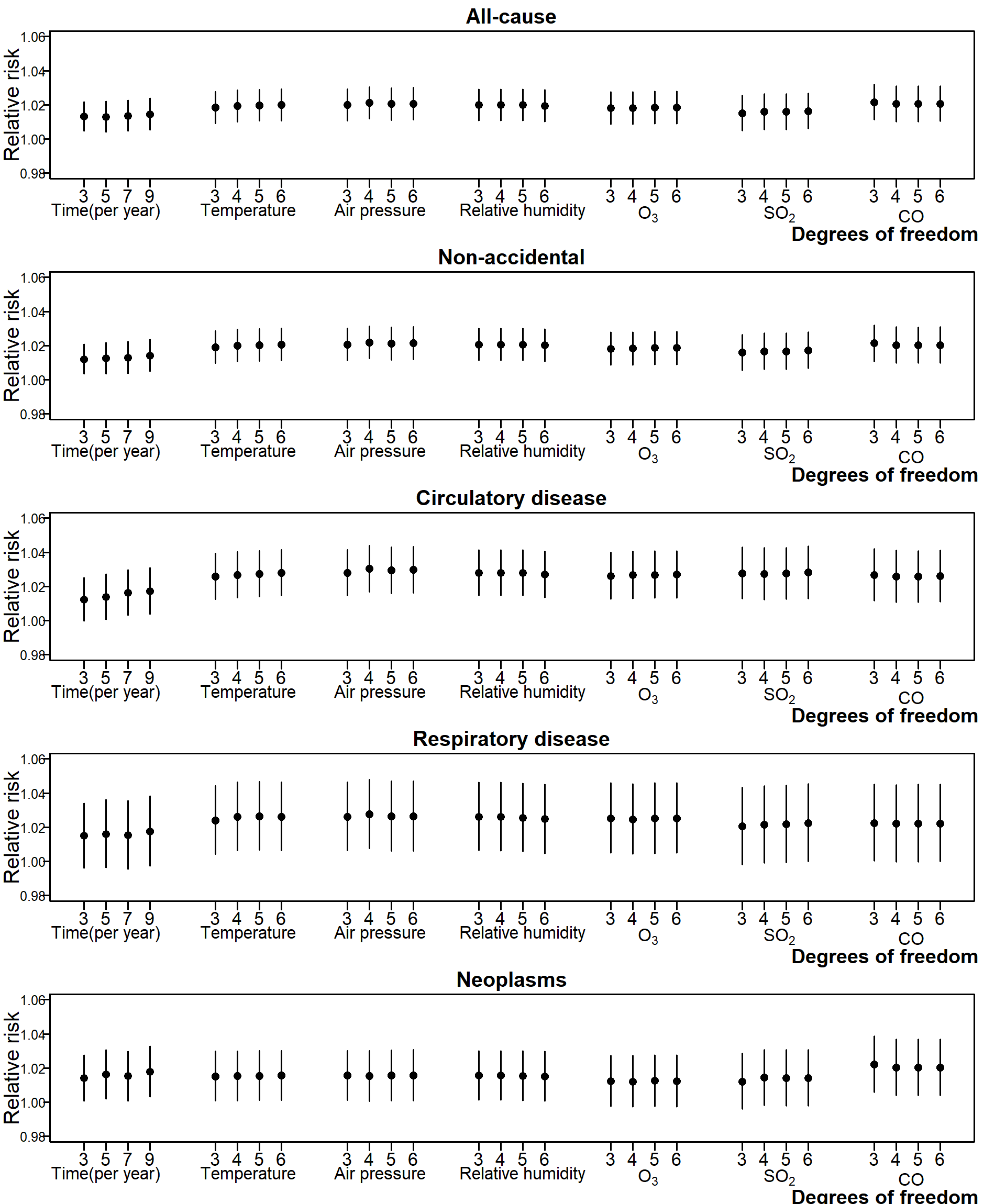


Fig. S5 Sensitivity analyses for cumulative relative risk (95%CI) of mortality associated with a 10 μg/m^3^ increase in PM_1_ at lag 0-3 day.


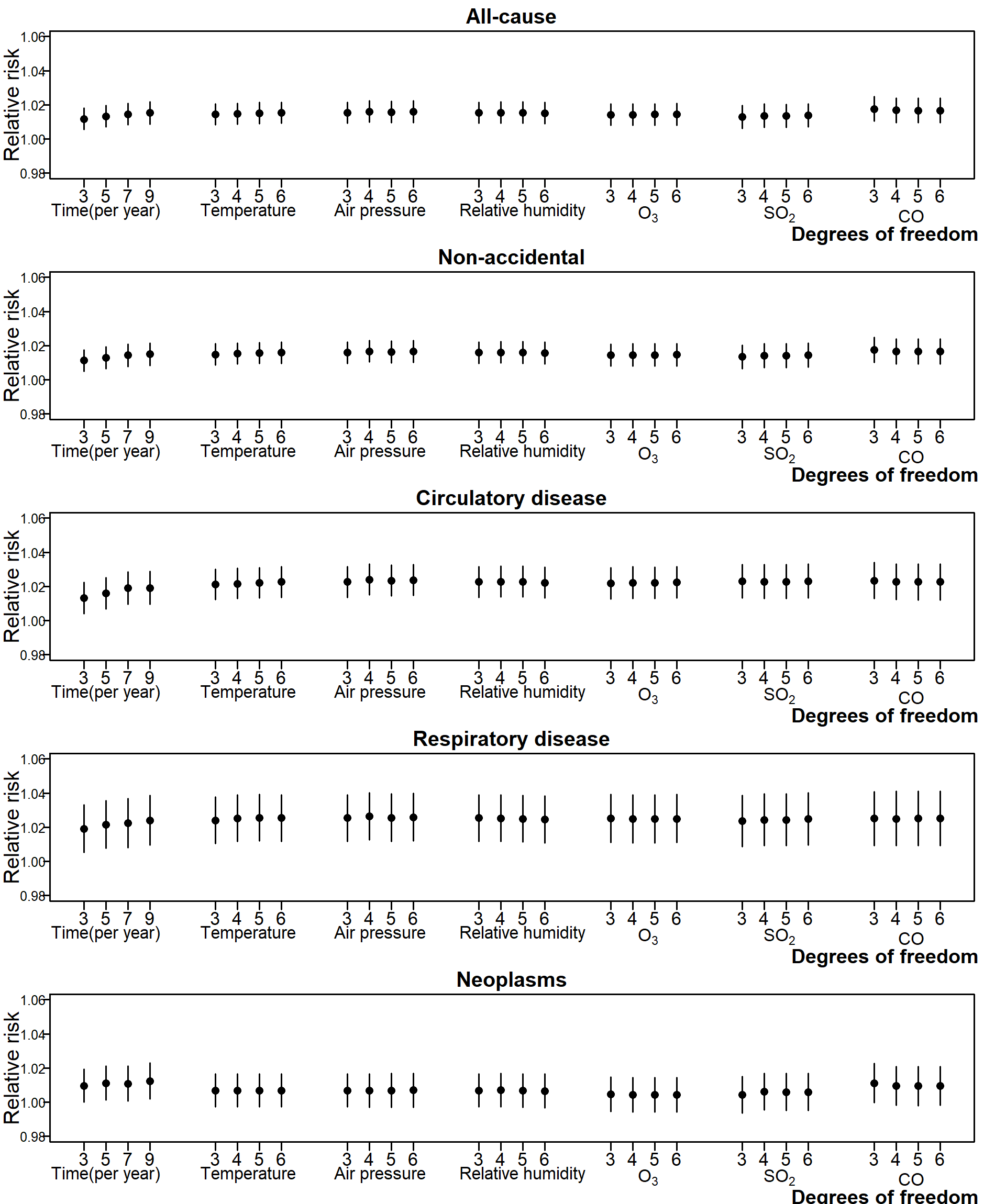


Fig. S6 Sensitivity analyses for cumulative relative risk (95%CI) of mortality associated with a 10 μg/m^3^ increase in PM_2.5_ at lag 0-3 day.


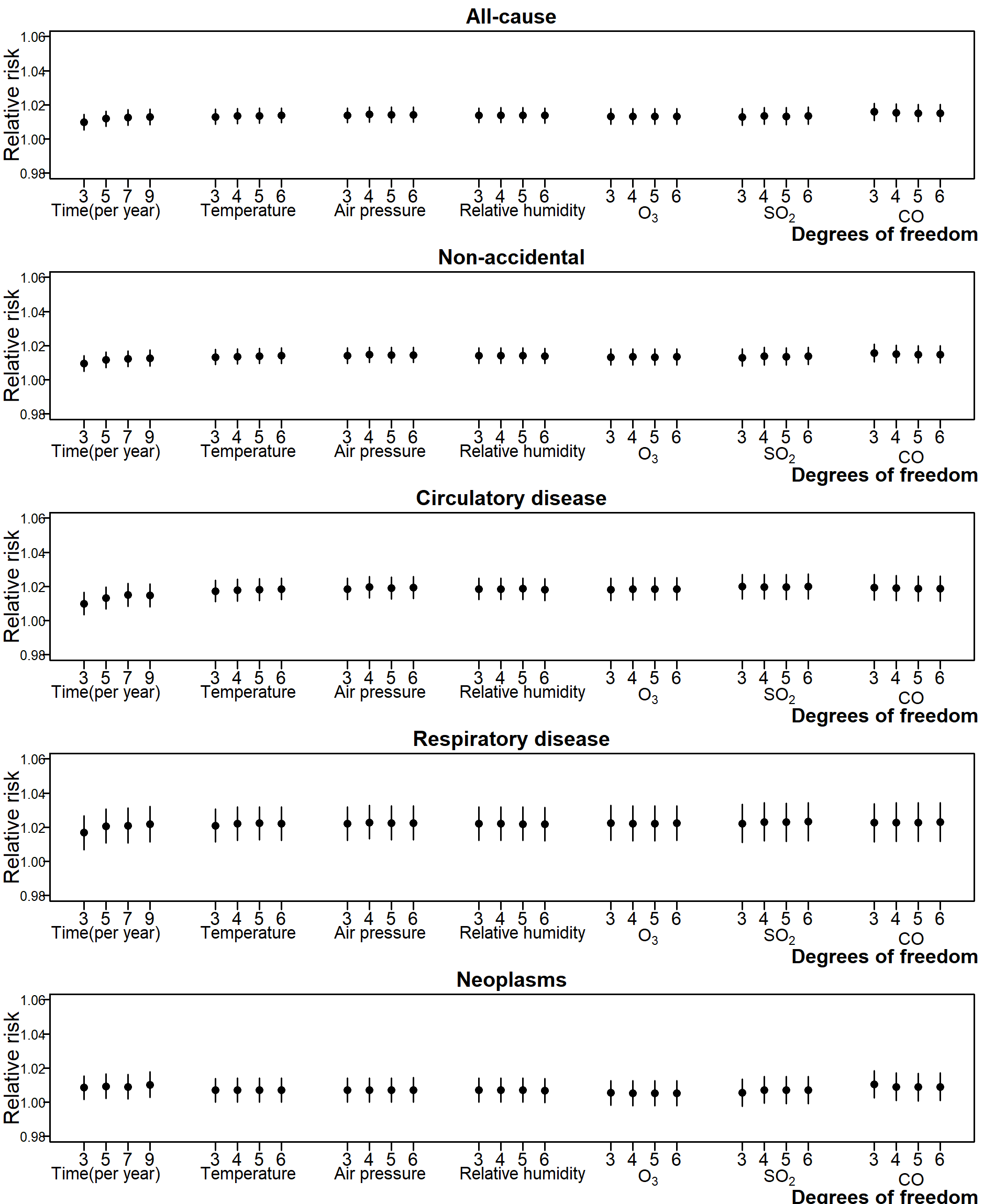


Fig. S7 Sensitivity analyses for cumulative relative risk (95%CI) of mortality associated with a 10 μg/m^3^ increase in PM_10_ at lag 0-3 day.
